# A critical review of recent trends, and a future perspective of optical spectroscopy as PAT in biopharmaceutical downstream processing

**DOI:** 10.1007/s00216-020-02407-z

**Published:** 2020-03-07

**Authors:** Laura Rolinger, Matthias Rüdt, Jürgen Hubbuch

**Affiliations:** Institute of Engineering in Life Sciences, Section IV: Biomolecular Separation Engineering, Karlsruhe Institute of Technology, Fritz-Haber-Weg 2, 76131, Karlsruhe, Germany

**Keywords:** Process analytical technology, Spectroscopy, Chemometrics, Downstream processing, Biologics

## Abstract

As competition in the biopharmaceutical market gets keener due to the market entry of biosimilars, process analytical technologies (PATs) play an important role for process automation and cost reduction. This article will give a general overview and address the recent innovations and applications of spectroscopic methods as PAT tools in the downstream processing of biologics. As data analysis strategies are a crucial part of PAT, the review discusses frequently used data analysis techniques and addresses data fusion methodologies as the combination of several sensors is moving forward in the field. The last chapter will give an outlook on the application of spectroscopic methods in combination with chemometrics and model predictive control (MPC) for downstream processes.

**Electronic supplementary material:**

The online version of this article (10.1007/s00216-020-02407-z) contains supplementary material, which is available to authorized users.

**Figure:**
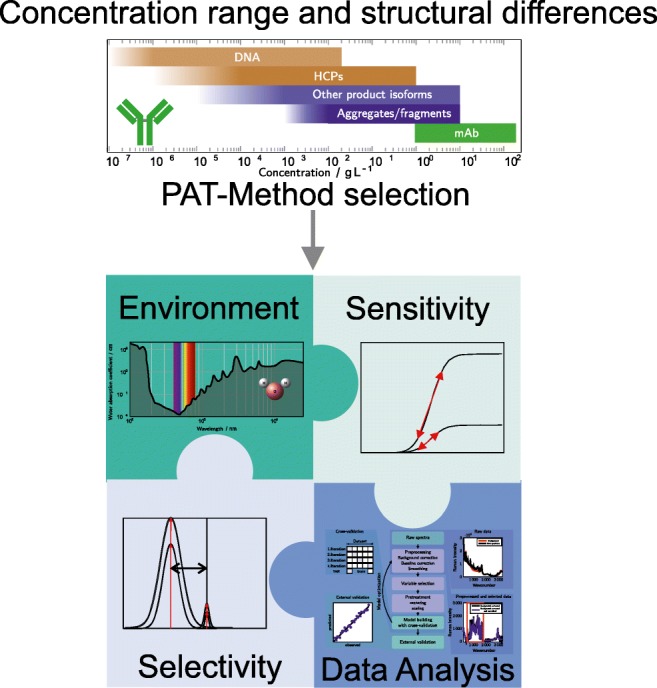
Graphical abstract

## Introduction

The biopharmaceutical industry currently faces major changes because of increasing competition in the field due to the market entry of biosimilars and increasing costs in research and development (R&D) of new drugs [1]. Since 1950, the number of approved drugs per billion US dollars spent for R&D has halved approximately every 9 years. This behavior is termed ’Eroom’s Law’ as it describes the opposite of ’Moore’s Law’ [2]. Not only are the costs per approved drug increasing, but the sales of off-patent blockbuster drugs are slowing down due to price competition from a variety of biosimilar products [3]. More companies seek to capitalize on the rapidly growing biologics market, which creates a competitive climate driving innovations for cheaper production, faster development, and improved quality of the biologics in order to gain a competitive edge [3, 4].

Digital transformation has already proven to drive the performance of companies in other industry sectors and has started to be adapted by the rather conservative biopharmaceutical industry as key strategy for production improvements as well [5, 6]. Part of the digital transformation of production processes are the implementation of appropriate measurement sensors and data analytics, i.e., process analytical technology (PAT), as information input for process control algorithms [6]. The achieved process control allows for optimal production runs and improves process robustness. The product quality may be improved by coping with process variability. Process robustness also shortens the development-to-market times, e.g., by facilitating scale-up, resulting in a competitive advantage [7].

While PAT has been successfully implemented as a pillar of process control for numerous small-molecule pharmaceuticals [8, 9], the high complexity of biopharmaceutical proteins and the close chemical similarity of contaminants impose a challenge for finding suitable PAT methods [10]. Ideally, a PAT method would be able to differentiate between product, process-related contaminants, and product-related contaminants in real-time. However, some product-related contaminants (such as subtle structural differences in oxidation or deamidation of single amino acids to the product) are detected by time-consuming analytical methods [11] e.g., analytical high-performance liquid chromatography (HPLC) methods, which typically take 30 min or more [12]. Larger structural differences (e.g., aggregation, misfolds, or PEGylated species) can be detected by on-line HPLC within 4 min to 6 min [13, 14], or by in-line spectroscopic methods in real-time [15, 16]. Here, spectroscopic methods offer several advantages over on-line PAT methods, such as rapid and automated detection with no sample preparation, conditioning, or destruction at comparable equipment costs [17]. However, one optical spectroscopic method alone offers a limited selectivity for the structural integrity of proteins [13], but optical spectroscopic methods can be easily combined with other spectroscopic or non-spectroscopic sensors to measure a large variety of attributes [18, 19]. Therefore, improved measurability and accuracy can be achieved by multiple sensors as compared to a single sensor [20, 21].

As the data complexity increases through the combination of multiple, possibly multivariate, spectroscopic sensors, advanced data analysis is required to extract information from the multivariate data about critical process parameters or critical quality attributes [22]. Data analysis from chemical data itself is also referred to as chemometrics [23]. Even though chemometrics generally covers the basic analysis from multiple data sources, data fusion methodologies are applied to chemical data for classification and prediction improvement [24]. As data analysis is often performed by software, the combination of sensors and data analysis for attribute estimation is often referred to as soft sensor [25].

Following this line of arguments, the section below will give a general overview and address the recent innovations and applications of optical spectroscopic methods as PAT tools in the downstream processing of biologics. This is meant as an addition to the comprehensive review by Rüdt et al. [13] in 2017. This review will focus only on optical spectroscopy, because other tools have been review in full elsewhere [26, 27]. As data analysis strategies are a crucial part of PAT especially for the interpretation of spectroscopic data, the third section will give a review about frequently used data analysis techniques and address data fusion methodologies as the combination of several sensors is moving forward in the field. The last section will give an outlook on the application of soft sensors (spectroscopic methods in combination with chemometrics) and model predictive control for downstream processes.

## Improvements in spectroscopy and applications

### Spectroscopic methods and their applicability to protein monitoring

The selection of appropriate techniques consisting of a spectroscopic method as well as a measurement setup is a key element in PAT [28]. The most important selection criteria are sensitivity and selectivity to evaluate the feasibility of the application. Other factors, like costs or complexity of the instrument, have to be evaluated for a successful process implementation in industry [17, 28]. In downstream processing of biologics, the dynamic range and measurement speed are important factors for the technology selection as well, because the concentration ranges are generally the largest in production and the feasible measurement times are the shortest.

**Fig. 1:**
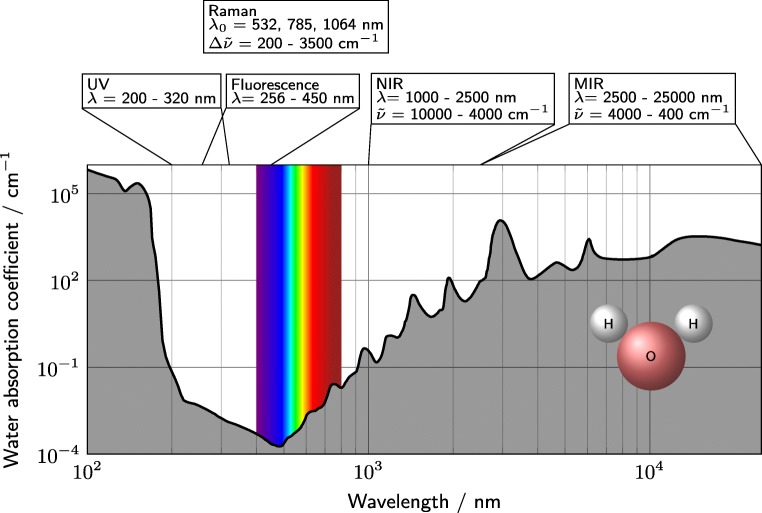
Typical wavelength ranges of UV, fluorescence, NIR, MIR, and Raman spectroscopy for the analysis of proteins are depicted. Additionally, the bulk water absorption coefficient is plotted over the wavelength to emphasize the effect of water on the different techniques. The visible spectrum is indicated for orientation. The data for the bulk absorption coefficient was taken from Segelstein [146]

The measurement environment (bulk solvent, temperature, pressure, etc.) greatly influences the sensitivity and selectivity of different methods. As the solvent often contributes the majority of molecules to the sample, it needs special consideration [28]. For biopharmaceutical processes, the solvent is in most cases water. Thus, high water signals are a typical problem in protein measurements. In Fig. 1, the bulk water absorption coefficients are depicted with reference wavelength regions for various spectroscopy types. Ultraviolet (UV) spectroscopy, intrinsic fluorescence, and often also Raman spectroscopy take place in regions of the electromagnetic spectrum with low water absorptivities. Even though near-infrared (NIR) and mid-infrared (MIR) measurements are generally thought of as selective and relatively sensitive, when it comes to measuring in water, these methods are impaired by the high water absorptivity caused by the OH band. In the NIR and MIR region, the water absorption spectrum dominates over the protein absorption (cf. Table 1). Additionally, the temperature sensitivity of the OH bands is a severe drawback for measuring aqueous solution in NIR and MIR, which makes tempered sample holders necessary [29].

**Table 1:** Molecular cross sections and extinction coefficients (if applicable) of IgG measured with different spectroscopic techniques

Spectroscopic method	Cross section *σ* as	Absorption/emission coefficient	Extinction coefficient water
	$-\log $(*σ* / (cm^2^ molecule))	/(l g ^− 1^ cm ^− 1^)	/cm^− 1^
UV (280 nm)	16	1.2 to 1.5 [143]	2.6 ⋅ 10^− 3^
Fluorescence	17	0.16 to 0.2 [68]	1.3 ⋅ 10^− 3^
NIR	16	1.2 [144]	25.6
MIR	15	12	1400
Raman (532 nm)	27	−	4.2 ⋅ 10^− 4^
Resonance Raman (229 nm)	25 [38]	−	6 ⋅ 10^− 3^
Rayleigh (633 nm)	18-19	−	3 ⋅ 10^− 3^

To compare different spectroscopic methods based on their sensitivity to proteins in water, the molecular cross sections, extinction coefficients, and the water absorption coefficients are listed in Table 1 for the different methods. The listed protein values are representative of an immunoglobulin G (IgG). Further information on the calculations are given in Supplementary Material Appendix A. Table 1 gives an overview on the sensitivity of the different spectroscopic methods by comparing the different scatter cross sections. However, it is important to consider the surrounding solvent water. It is beneficial to achieve a high ratio of protein scatter cross section to water absorption. Table 2 gives an overview which protein structural elements are measurable with different spectroscopic methods. Table 2 helps to evaluate, whether the protein structure feature of interest can measured with the selected spectroscopic method. Table 1 provides a lead on the measurability of a certain protein concentration in water with the selected spectroscopic method. Generally, it is important to look at the protein and water absorption in the wavelength range of a spectroscopic method to draw the right conclusions.

**Table 2:** Structural elements of proteins observed with different spectroscopic methods

Spectroscopic method	Relevant structural elements
UV	Aromatic amino acids, peptide bonds, disulfide bridges, size (light scattering)
Fluorescence	Aromatic amino acids
NIR and MIR	Peptide bonds
Raman	Aromatic amino acids, peptide bonds, disulfide bridges
Resonance Raman	Excitation ≤ 220 nm: peptide bonds
	Excitation ≥ 229 nm: aromatic amino acids
Rayleigh	Protein weight and shape

The information was compiled from [35] and [145]

In the NIR and MIR regions, proteins show high absorption coefficients compared to the other methods due to the strong absorption of the C=O bond [30]. However, since water absorption in this region can be a 100-fold higher for dilute concentration, NIR and MIR are not well suited for quantifications down to 1 g l^− 1^ [31], which means that the quantification of contaminants in the process will be challenging due to the low concentrations. In contrast, UV and intrinsic fluorescence spectroscopy show little water interference, but absorption and emission coefficients comparable to those in the NIR and MIR regions. Therefore, quantification of proteins in the mg l^− 1^ range is possible with UV and fluorescence spectroscopy [32]. Rarely, there are deviations from the Beer–Lambert law due to, e.g., adsorption to the measurement cell walls, which can impair the quantification limits [33]. Even though intrinsic fluorescence spectroscopy can quantify proteins to the mg l^− 1^ range, it behaves only linearly at low concentrations (absorbance below 0.05) due to the so-called inner filtering effect. The inner filtering effect is caused by light absorption in the sample and results in distorted emission intensities and spectra, which cause a nonlinearity between fluorescence intensity and protein concentration [34, 35]. Consequently, UV spectroscopy typically offers a greater linear range than fluorescence spectroscopy [36].

Like UV and intrinsic fluorescence spectroscopy, Raman spectroscopy usually has very low water interference as well [29] but, due to very small protein scattering cross-sections (cf. Table 1), the water bands are dominant for dilute protein solutions. Therefore, protein structure studies often utilize the resonance enhancement effect in the UV range [37] to increase the intensity of protein bands and take advantage of the low water absorptivity in the UV. The resonant effect of the Raman scattering in the UV region, referred to as UV resonance Raman (UVRR), is caused by the absorption of aromatic amino acids or the polypeptide backbone of proteins. The Raman cross section of the modes coupled to these resonant electronic transitions can increase by a magnitude of five [38]. Besides the enhancement advantages of UVRR, there are some drawbacks like photodamage due to exposure to UV light or a loss of linearity between the signal intensity and the concentration of protein due to the reabsorption of photons [31]. This effect is comparable to the inner filter effect observed in fluorescence measurements [31].

Not only does the broad concentration range during purification of biologics impose a challenge on the linear range and sensitivity of analytical methods but the complexity and chemical similarity of contaminants to the respective product call for a high level of selectivity for quantification as well [12, 39]. The International Union of Pure and Applied Chemistry (IUPAC) defines selectivity as “the quantitative characterization of a systematic error in the measure of a signal caused by the presence of concomitants in a sample” [40]. In other words, it is the accuracy of quantifying an analyte in a mixture [41]. For spectroscopy, this implies that the signal/bands of interferent and analyte need to be distinguishable for a high selectivity [42]. UV spectroscopy observes the electronic state transitions. The most prevalent chromophores in proteins are the peptide backbone, the aromatic amino acids (tryptophan, tyrosine, and phenylalanine), and disulfide bridges formed by oxidation of two cysteine residues to cystine [35, 43]. Furthermore, UV spectra contain information on protein folding (via wavelength shifts of the involved chromophores) to aggregation (via light scattering), even though these different energy states overlap to the broad electronic absorption spectra usually observed in solution [35]. This information can be used in combination with multivariate data analysis tools, like partial least squares (PLS) models, to deconvolute several species, which has been shown in several case studies [15, 43–46].

In MIR, up to nine characteristic bands can be observed for proteins, namely and in order of decreasing wavenumber amide A, B, and I to VII [47]. The amide I band (1610 cm^− 1^ to 1700 cm^− 1^, mostly C=O stretching) and amide II (1480 cm^− 1^ 1575 cm^− 1^ N-H bending and C-N stretching) are most pronounced. These bonds are influenced by the hydrogen bonds around them, formed by the folding of secondary structure elements [48]. Aromatic amino acids absorb as well, but mainly in the spectral region of the amide I band from 1610 cm^− 1^ 1700 cm^− 1^ [49]. Due to the overlapping absorptions, highly convoluted and similar spectra are observed for proteins. However, MIR spectroscopy can be used to distinguish between proteins and other substances used by the biopharmaceutical industry, like polyethylene glycol (PEG) or Triton-X [16, 50]. These measurements were carried out with fourier transform infrared spectroscopy (FTIR), which is not entirely suitable for processes due to moving parts and vibrational sensitivity [51].

NIR spectroscopy has the advantage of having no moving parts. However, the selectivity is generally low, due to the superposition of different overtones and combination bands in the NIR region [52].

As a complementary vibrational spectroscopic method to MIR, Raman spectroscopy provides similar information on the secondary structure of proteins. Similar to MIR, the amide bands (especially amid I and III) are strong in Raman spectra [35]. Additionally, Raman offers more structural details on aromatic amino acids and disulfide bonds that reflect the protein tertiary structure. These information can be observed because some molecular groups in the protein side chains, such as C=C, C-C, S-S, C-S, S-H groups, have large polarizabilities which results in large Raman activities [17]. In contrast to MIR, these bands generally overlap less with the amide bands [53] and, therefore, the selectivity of Raman for proteins is generally higher. Furthermore, as discussed above, the impact of the bulk water is smaller for Raman spectroscopy.

The selectivity can be improved by chemometric methods, also referred to as computational selectivity [41], which will be further addressed in “Advanced data analysis and machine learning”. The initial selectivity of a sensor is, however, an important driver of the computational selectivity [54]. This might be the reason why UV spectroscopy in combination with chemometric methods has successfully been applied to a wide variety of problems in the last decade [13] as a result of its strong sensitivity and decent selectivity. Raman spectroscopy is frequently applied in upstream processing in research and industry due to its high selectivity and low water interference [55] despite the relatively long measurement times. Instrumental innovations shorten measurement times and make Raman spectroscopy more amendable for downstream processing as well. New applications of UV, fluorescence, Raman, and multimodal spectroscopy as PAT tools for downstream processing will be addressed in the following subsections in detail.

### UV spectroscopy

A challenge of UV spectroscopy is the limited linear range of the instruments [13]. The application of variable pathlength (VP) UV spectroscopy allows for concentration measurements in an extended dynamic range. The necessary equipment has been commercialized and is available under the brand names FlowVPE and SoloVPE [56, 57]. Recent applications of VP UV spectroscopy showed the applicability to a monoclonal antibody (mAb) chromatography step from 0 g l^− 1^ to 80 g l^− 1^ [15] and to an ultrafiltration/dialfiltration (UF/DF) process with a range from 2.8 g l^− 1^ to 120 g l^− 1^ [58]. For most flow rates, the FlowVPE can be used in-line. Due to the used monochromator, the FlowVPE takes a significant amount of time (typically ≥ 30 s) [15] to collect a full spectrum. Replacing the monochromator with a polychromator and a diode array detector could improve measurement time in the future and reduce the number of moving parts in the VP spectroscopy system.

Alternatively, the use of attenuated total reflection (ATR) flow cells could be of interest for measuring UV spectra in high concentration protein solutions. However, to the best of our knowledge, no studies with a focus on biologics have been published using UV ATR flow cells.

### Fluorescence spectroscopy

Pathak et al. demonstrated that the fouling of Protein A resin can be observed by diffuse transmission fluorescence spectroscopy [59]. While it is interesting that the fluorescence increases due to protein fouling on the resin, a direct correlation is difficult. Due to the setup path length of 1 cm, the study is not directly applicable for industrial scale. Higher path lengths might result in a more pronounced inner filter effect and nonlinearities. Additionally, Zhang et al. [60] showed that the resin fouling is not homogeneous over the column, which makes multiple measurements necessary to provide a holistic picture over the column.

### Raman spectroscopy

In general, Raman scattering is a weak effect because only about 1 in 10^10^ photons undergoes Raman scattering in aqueous protein solutions [61]. To set this into perspective with absorption experiments where a mAb (*𝜖* = 14 l g^− 1^ cm^− 1^) will absorb around 90% of the incident photons over 1 cm cuvette at a concentration of 0.7 g l^− 1^ [61]. The low scattering cross section explains why the first Raman scattering measurements took days [62]. Due to the development of compact and high power lasers, charge-coupled devices, fiber-optics probes, and further optical component enhancements, measurements can be realized in minutes today because of the increased photon output and collection efficiency [63, 64]. With standard Raman analyzers, measurement times of 12.5 min (785 nm excitation, 75 s collection time with ten exposures) [65, 66] are frequently applied to upstream processes. As upstream processes can take a couple of weeks [67], a measurement time of 12.5 min is sufficient. However, for downstream process units with operation times of a few hours [67], measurements need to be significantly faster. Usually, 30 s is considered near real-time in downstream processing [15].

There are several factors influencing the strength of the Raman signal and hence the measurement speed, but all of them rely either on increasing the amount of scattered photons or converting more scattered photons to a signal. The Raman efficiency increases by a fourth-order function as the laser frequency is decreased. Hence, the shorter the laser wavelength, the more intense is the Raman signal [63]. Unfortunately, a shorter wavelength does not always result in a better Raman spectrum because fluorescence can overshadow the Raman signal. At the very least, a laser excitation wavelength and according Raman scattering range outside the intrinsic fluorescence range of proteins from 257 nm to 450 nm [68] should be chosen for the downstream process to avoid fluorescence overpowering the Raman signal. This is assuming that other potential fluorophores, like phenol red from the cell culture medium [69], which fluorescence outside the intrinsic protein fluorescence range, are not present. At a laser excitation wavelength below the intrinsic protein fluorescence range, e.g., 254 nm, there is no interference from fluorescence. While it might be difficult to apply standard laser emission wavelengths, like 532 nm or even 785 nm, to upstream processes due to fluorophores in cell culture media, these wavelengths can usually be utilized for downstream processing.

Besides lowering the excitation wavelength, the Raman signal intensity can be enhanced by increasing the laser power, increasing the interaction length between the laser and the sample by multiple-pass arrangements [70], or increasing the collected light through sample optics with reduced photon losses in the spectrometer [71].

Feidl et al. [72] made a multi-pass flow cell by using a concave mirror behind a cuvette to increase the signal to monitor the breakthrough of a Protein-A column. Even though this is the first application of Raman spectroscopy to downstream processing, the publication shows that advanced chemometrics and a significant computational effort were necessary to reach a model that is comparable to UV spectroscopy combined with a basic PLS model [73]. It is worth noting that the obtained Raman spectra were dominated by water. Therefore, it might be possible that the displacement of water due to an overall increase in protein concentration may be important for the underlying correlation.

### Multimodal spectroscopy

As outlined by Rüdt et al. [13], one sensor alone will not be able to measure every product quality attribute during production. Even for measuring one quality attribute, the combination of multiple sensors might be necessary. For example, the real-time monitoring of the mean molecular weight during a flow-through hydrophobic interaction chromatography (HIC) step for a mAb has been realized by static light scattering and concentration measurements by UV spectroscopy [74]. Because the scattered-light intensity is not only influenced by the molecular weight but by the concentration as well, a concentration measurement is necessary to calculate the molecular weight. Based on the calculated mean molecular weight signal, the flow-through step was terminated after a 1.5 % dimer breakthrough. It should be mentioned that this setup is limited to near-isocratic buffer conditions. For, e.g., cation exchange chromatography (CEX) with high- and low-salt conditions and therefore a changing refractive index, additional sensors, like a refractometer, might be necessary for accurate quantification.

Another application of light scattering is the downstream process of virus-like particles (VLPs). Rüdt et al. monitored the diafiltration reassembly steps of three different VLP constructs at different conditions with UV spectroscopy and light scattering [46]. The scattered-light intensity was correlated to the assembly progress and UV spectroscopy provided information on the concentration of the VLPs as well as the rate of the assembly due to changes in the local environment of tyrosine residues.

Another approach, besides calculating the attributes of interest from different sensors by physically founded equations, is to fuse all data for statistical model building. This approach was applied by Walch et al. [18], where fusing data from seven sensors lead to a total of 15,725 input variables. These input variables were then used for PLS model building to predict antibody concentration, high molecular weight species (HMWS), deoxyribonucleic acid (DNA), host cell protein (HCP), and monomer content by PLS regression. It is important to note that such an approach can lead to physically unrealistic results. In the study, the pH was used in a PLS model to predict the mAb concentration. PLS modeling is a linear regression approach that can only handle nonlinearities to a point, where a linear approximation of a nonlinear problem is feasible. A logarithmic pH value might not be a meaningful input for a linear regression model without a variable transformation. Similarly, ratios, like HMWS, DNA, or HCP content, as output values should be handled with care as they are not linearly related to unscaled spectroscopic data. In a small range, where the relationship between the ratio and the spectral data can be linearly approximated, the use of PLS models is feasible [75, 76]. For strong nonlinearities, nonlinear methods, like nonlinear PLS models [77] or artificial neural networks (ANNs) [78], should be considered. In our opinion, for the prediction of ratios with values covering several orders of magnitude (i.e., DNA content, and HCP content) nonlinear methods should be used. Based on the data published by Walch et al., it cannot be precluded either that the PLS models rather correlate the DNA and HCP content to the inverse of the protein concentration than being based on an actual causal relationship. Therefore, these PLS models might only work in a limited design space, where every run has the same trends and the DNA and HCP concentration in the eluate is constant. Then, the DNA and HCP concentrations per part of mAb are only influenced by the mAb concentration and could be well predicted to unrealistic concentration limits for optical spectroscopy. Additionally, if a large number of input variables and only a small number of samples is available, spurious correlations between two data sets are likely to occur when variable selection is done even while using cross-validation (CV) [79].

Sauer et al. [19] used the same experimental setup as Walch et al. [18] but chose to use the statistical framework of STructured Additive Regression (STAR), which provides means to include a wide range of nonlinear effects into model building, e.g., by including bivariate interaction terms [80]. However, the authors chose to exclude bivariate interaction terms for all spectroscopy sensors due to the required computational power. Therefore, it remains unclear how the model structure reflects the nonlinear response of, e.g., the DNA and HCP to mAb concentration. The additional degrees of freedom do not only affect the computational demand during calibration; during validation, it also becomes far more challenging to assert that the model does not overfit compared to purely linear models.

When using multiple sensors in a process stream, it is important to account for dispersion between the detectors. Especially for lab-scale chromatographic setups, the peak will change its shape as the detectors are passed and time alignment alone might not be sufficient to overlay the signal of the different sensors. Here, proper data treatment and analysis are important to draw the right conclusions which will be discussed in the next chapter.

## Advanced data analysis and machine learning

**Fig. 2:**
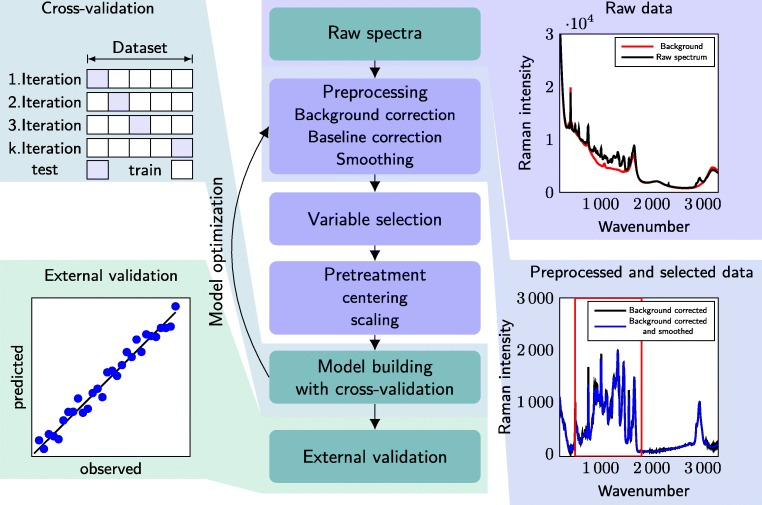
General workflow for PLS model building. More information on the different steps of the workflow are provided in “Advanced data analysis and machine learning”

Machine learning refers to different algorithms to develop models for pattern recognition, classification, and prediction derived from existing data [81]. PLS models and its variations are the most frequently used machine learning methods for multivariate data analysis (MVDA) of spectral data in bioprocesses [82, 83]. In Fig. 2, a general workflow for model building is depicted with illustrations from Raman spectral data for concentration determination as example. Generally, model building starts by choosing the design space for the model and recording spectral data. Subsequently, spectra are preprocessed, outliers are removed, and the data are pretreated to improve data quality. Model building may include CV and model optimization until the optimal model is found. Before productive use, it is compulsory to evaluate the model performance with an external data set as it has been shown that internal validation is not sufficient [84]. All necessary steps to obtain a valid model are discussed in more detail in the following section.

### Sample selection

Generally, it is advisable to choose samples that are representative of the purpose of the model [85]. Therefore, known process variations should be included into the model. This could be done, for example, by recording different runs with variations in the normal operating ranges, like different batches, upper and lower limits for buffer composition, and load density of chromatography columns, etc. If there are no restrictions on the compositions of the samples, the use of a D-optimal design for a design of experiment (doe) approach is applicable to the distribution of samples in the design space [86]. Regarding the minimal sample size required for PLS calibration, rough heuristic rules advocate at least five or ten samples per adaptive parameter, i.e., latent variables [87–89]. Generally, it is not possible to choose more latent variables than calibration samples, as this is a restriction of the algorithm. PLS models with as many latent variables as samples will be without doubt over-fitted. Depending on the data complexity, PLS models for spectroscopic data can even have around ten latent variables without over-fitting [90, 91]. The data set is split into calibration and external validation test set at a ratio of 2/3 to 3/4 in terms of calibration samples to the sample size of the data set [86]. The exact ratio depends on the sample size of the data set [86]. For smaller data sets with fewer samples, a higher ratio of calibration samples to available samples is chosen. To ensure a uniform distribution of calibration and validation samples over the design space, a supervised sample selection such as the Kennard–Stone algorithm, is preferred compared to random sampling [92].

### Preprocessing

The objective of the preprocessing of spectral data is to remove extraneous variance, such that the data adheres closer to the Beer–Lambert law [93]. Depending on the spectroscopy method, different preprocessing steps are required to reach this objective [94]. A review on preprocessing for Raman and FTIR is given by Gautam et al. [94]. For UV, 2D fluorescence, and light scattering usually no extensive preprocessing, except for the background correction, is necessary.

Often, the spectrometer software and correct calibration of the instrument remove instrument- or method-specific effects, such as detector nonlinearities, wavelength shifts, or interfering signals. Especially for Raman spectrometers, instrument calibration is necessary due to possible shifts in the laser excitation wavelength. Therefore, Raman spectrometers are generally calibrated with external light sources and reference substances to calibrate *x*- and *y*-axis and the laser wavelength [95]. Usually, cosmic rays are already removed before preprocessing begins.

The most common preprocessing steps for UV, NIR, MIR, fluorescence, and Raman spectroscopy include smoothing as well as baseline, background, and scatter correction [96]. Background correction procedures minimize the effect of a varying background caused by fluorescence, if applicable, of the sample or thermal fluctuations on the detector [97] and the buffer contribution to the spectrum for dissolved samples. Usually, if the background correction corrects for drifts of the spectrometer, no additional baseline correction is necessary. However, if a baseline correction is necessary, de-trending, asymmetric least squares smoothing (ALS), or derivations [96] could be used. De-trending relies on fitting a polynomial to the spectrum and subtracting it from the spectrum while ALS involves an inert estimation of the background by an asymmetric least-squares fit. First-order derivatives eliminate a constant offset while second-order derivatives remove a constant offset and slope. Because derivatives make high-frequency noise more pronounced, Savitzky–Golay filters are often used to smooth and derive [93, 96]. However, Savitzky–Golay derivations are also prone to high-frequency noise, depending on the window width. High-frequency noise can influence the model and cause overfitting [93]. Therefore, (extended) multiplicative signal correction (MSC) is generally recommended as preprocessing technique [93, 96, 98]. In practice, derivatives are still frequently used due to their simplicity and ease of use. For solely smoothing data, Savitzky–Golay filters are still the most used smoothers due to their superior preservation of peak shapes compared to e.g., the moving average filter [99].

For scatter correction, the MSC algorithm was developed by Martens et al. [100]. MSC uses a blank spectrum as reference, if available, or a mean of all recorded spectra to estimate correction coefficients for the spectra. Later on, the MSC algorithm was expanded to include the wavelength dependency of the scattering intensity and corrections for known spectra, referred to as extended multiplicative signal correction (EMSC). This caused the development of other de-trending techniques, like orthogonal signal correction (OSC), orthogonal PLS (O-PLS) [93]. The use of MSC or related techniques can reduce the number of latent variables in a PLS model and enhance the chemical information in the spectra to facilitate interpretation [99]. Additionally, the EMSC can normalize the spectra. However, normalization of spectra removes absolute concentration information and is therefore not recommended for concentration-dependent applications.

Generally, it is worth to keep in mind that preprocessing may also remove useful information (e.g., fine structures in the spectra, informative scattering effects) [98]. Therefore, it is sometimes beneficial to preprocess data less in order to preserve most information.

### Outlier detection

Proper handling of outliers is essential for data analysis because outliers introduce large variance to the model which can disturb the model [79]. Principal component analysis (PCA) is a useful tool to look at the variance of the data to evaluate whether it is an unusual variance in the model plane or outside of the model plane. A common way to remove outliers within the model plane is to look whether samples lie outside of the 95*%* confidence limit of the *Hotelling’sT*^2^ ellipse in the PCA *t*_*i*_ vs. *t*_*j*≠*i*_ score plots for each score to another [101]. The ellipse shows the distance from the origin in the model plane with the chosen confidence. Additionally, outliers outside the model plane can be evaluated by calculating the distance of an observation in the training set to the model hyperplane [91] or by calculating the residuals of the observations [17].

As PCA reflects the main variations in the *X*-data, the results of a PCA-based outlier detection might be misleading if the main variations in the data is not correlated to the *Y* -variables [85]. As the purpose of preprocessing is to remove variance outside of the Beer–Lambert law, the main variance in the *X*-data should be correlated to the *Y* -variables. Outliers due to erroneous measurement should be removed before variable selection. Outliers with a large variance in the model should be either removed during sample selection due to the irrelevance to the model or be included as important process variance. However, outlier detection was not included in the general workflow for PLS model building depicted in Fig. 2, because it can be part of sample selection with manual inspection of the spectra for erroneous measurements or take place before model optimization.

If in doubt whether to remove an outlier or not, it is useful to compare the models before and after removal. If the model changes dramatically, e.g., in the amount of latent variables, scores, etc., the outlier removal is important. Otherwise, the sample can be included [101].

Generally, outlier detection and removal can be automized, but it is important to point out the risk of automatic outlier removal. Outliers may carry valuable information about the system and process. For instance, the ozone hole could have been detected earlier if it had not been for automatic outlier detection methods [102]. In context with optical spectroscopy in processes, outliers indicate unusual disturbances of the spectrum. Here, outliers could be used to detect process failures, e.g., equipment failures or air entrapment.

A more extensive overview of outlier removal is given by Hadi et. al. [103].

### Variable selection

PLS models and the corresponding conclusions can be highly dependent on the included *X*-variables [85]. Even though weighting of the *X*-variables according to the information content for the prediction of a univariate *y*-variable is an inherent property of the PLS algorithms, the inclusion of irrelevant and noisy variables can increase the prediction error of the PLS models [104]. Therefore, areas in the spectrum with high variance, but little to no correlation to the chemical properties of the sample, and areas containing only noise should be left out of the model to improve the prediction ability [79]. Further exclusion of *X*-variables can still improve the prediction ability of the model but the model robustness can decrease due to the increased risk of over-fitting by choosing less causal *X*-variables but with a higher correlation to *y* [79, 85]. Andersen et al. [79] showed that variable selection can lead to a statistically significant correlation of random *X*-data to a *y*-variable for more *X*-variables than samples even when using CV. Therefore, a comparison between selected variables and variables known for containing the desired information on the chemical or physical behavior of the system is important to prevent over-fitting and can give more insight into the data. A review of variable selection techniques would go beyond the scope of this manuscript. However, reviews about various variable selection methods for spectral data are given by Anderson et al. [79] or Mehmoood et al. [104].

### Pretreatment

Data pretreatment strategies focus on the relation between different samples in one variables (i.e., column vectors), in contrast to preprocessing, which focuses on the different variables from one sample. Sometimes, pretreatment techniques are also referred to as preprocessing. In our opinion, distinct terms should be used to emphasize the underlying differences. Next to the already-mentioned difference regarding to which matrix dimension the methods are applied (i.e., applied variable/block-wise versus in the spectral direction), it is also worth noting that data pretreatment is not limited to the *X*-data but can also be applied to the *Y* -data. Importantly, the pretreated values will change when samples are removed from the calibration set, while the preprocessed values stay the same.

Centering, scaling, or variable transformations are used as most common pretreatment techniques [105]. Mean-centering is often applied to data that is obtained with a single instrument, as all variables are defined with the same unit [91]. Centering may improve the numerical stability and interpretability of the results, as the model is focused on explaining data variance rather than data magnitude [105, 106].

Scaling methods divide each column vector by a different factor, e.g., to give each column vector a unit variance [91]. The goal of scaling is to reduce the influence of large numeric values in order to focus on correlating the *X*- to the *Y* -variables. Pretreatment is especially important if variables are measured by different sensors, as this may result in variables with different scales. Models, such as PLS and PCA, often try to explain the largest covariance in data, which is bias to variables with the largest numerical values [91]. There are a plethora of different scaling techniques to account for different effects [105], which is important for handling multiple differently scaled variables. This topic will be discussed further in “Data fusion”.

Transformations are necessary if the numeric values of *X*-variables are not linearly correlated to the *Y* -variables for linear modeling. This can be important to e.g., diffuse reflectance intensities or pH values.

### Model building and model optimization

An important point during model building is to select the correct model type, when having multiple *Y* -variables. For spectral data where the *Y* -data (e.g., concentrations of multiple components) are not correlated, it is useful to make a PLS model for each component, also referred to as PLS1-models [99, 106].

During model building, it is essential to determine the correct number of latent variables for the PLS model, also referred to as model complexity. Due to numerous and collinear *X*-variables, there is a substantial risk of overfitting the model. Overfitting occurs, when added latent variables only fit random noise, which results in a loss of the predictive power. CV has proven to be a useful tool for determining the influence of latent variables on model performance and reducing the possibility of random correlations [106, 107].

To perform CV, the data set is divided into multiple subsets (between five to nine [108]), and PLS models are formed for a given number of latent variables until every subset has been left out once. Subsequently, the sum of squared differences between experimental and predicted *Y* -values is calculated for the left-out data for all computed models to estimate the predictive ability, or goodness of prediction *Q*^2^, of the model. The number of latent variables is set to the lowest number where adding another variable does not significantly increase the predictive ability [91, 106].

Besides the number of latent variables, data preprocessing and variable selection are other approaches that can be optimized in order to obtain an improved PLS model [109]. Preprocessing and variable selection usually rely on experience and manual inspection of the samples, where a certain preprocessing algorithm and windows of the spectra are selected. While this improves the performance of the PLS model, it is often not intuitive to find the best combination of all optimizable parameters [79]. Therefore, the use of a parallel genetic algorithm (GA) can be useful to find the optimal PLS model [110] to optimize the preprocessing and variable selection in one algorithm. However, since GA are prone to overfitting, it is important to use multiple GA runs and set the optimization parameters, e.g., window size, properly [111]. A comprehensible review on variable selection techniques was published by Andersen et al. [79].

A different approach for model optimization is used by Feidl et al. [72] and Narayanan et el. [112], where all useful combinations of preprocessing, pretreatment, outlier removal, smoothing, and variable selection were calculated and the best preprocessing and pretreatment method was chosen judged by the decrease in root mean-square error of cross-validation (RMSECV) and root mean-square error of prediction (RMSEP).

In this case, the RMSECV and RMSEP indicated the same optimized preprocessing an pretreatment method. Therefore the model optimization was not influenced by the RMSEP. Nevertheless, it is important to note that models must not be optimized by use of the RMSEP. It is counterproductive to use the same key figure for optimization and evaluation of the model, because the model is then optimized to give the lowest RMSEP and not to find an actual correlation.

### Model validation

The goal of model validation is to ensure the quality of the prediction in terms of a causal and robust correlation [17]. There are several key figures to evaluate models [91, 99]. The root mean-square error (RMSE) is the predicted residual error sum of squares (PRESS) divided by the sample size *n*, see Eq. 2. For the calculation of the PRESS with Eq. 1, *y*_*i*_ is the measured value and $\hat {y}_{i}$ is the predicted value. The difference between RMSECV and RMSEP is the used data to calculate the error. In case of the RMSECV, it is the RMSE of the samples that were left out in the CV step, also known as internal validation. In case of the RMSEP, the samples from an external validation sets are used.

1 $$ PRESS = \sum\limits_{n=1}^{N} (y_{i} - \hat{y}_{i})^{2}, $$

2 $$ RMSE = \sqrt{\frac{PRESS}{n}} = \sqrt{\frac{{\sum}_{n=1}^{N} (y_{i} - \hat{y}_{i})^{2}}{n}}. $$

Especially for small data sets, the RMSECV and RMSEP depend heavily on the used samples. Therefore, when comparing different PLS models with the same data set, the same samples should be used for calibration and validation, respectively. For comparison of different PLS models with different data sets, it is useful to evaluate the model by the coefficient of determination for the calibration *R*^2^ after Eq. 3, where $\bar {y}$ is the mean of *y*. The coefficient of determination for the CV *Q*^2^ is calculated after Eq. 3 as well for the left-out samples during CV. It should be noted that the difference between *R*^2^ and *Q*^2^ are the samples used for calculation. *R*^2^, also referred to as *R*^2^*Y* is the variation of the *Y* -variables explained by the model. *Q*^2^, also referred to as *Q*^2^*Y*, is the variation of the *Y* -variables predicted by the model. It should be noted that as a replacement for the RMSEP, the $Q^{2}_{\text {ext}}$ calculated with the external validation set used for the RMSEP calculation can be used as well to give a more representative key figure for the prediction ability on an external validation set [91].

3 $$ R^{2}= 1-\frac{PRESS}{{\sum}_{n=1}^{N} (y_{i} - \bar y)^{2}} = \frac{{\sum}_{n=1}^{N} (y_{i} - \hat{y}_{i})^{2}}{{\sum}_{n=1}^{N} (y_{i} - \bar y)^{2}} $$

While statistic methods try to establish a correlation between *X*- and *Y* -variables, it is important to emphasize that this correlation might not necessarily be a causal relation [83, 85, 99]. Even if model building was successful, a spurious correlation or an indirect correlation possibly may have been found. Indirect correlations can sometimes be used to quantify a component A, if, e.g., actually component B is measured, but is converted into component A at a fixed ratio [113]. Even in this case it is useful to be aware of this indirect correlation to draw the right conclusions from the model. Indirect and spurious correlation have been widely discussed for quantitative structure–activity relationship (QSAR) models, because QSAR models can be prone to these kinds of correlation due to the vast amount of *X*-variables, which make it possible to almost always find some kind of correlation. For verification of meaningful correlations, Wold et al. [108] published a method consisting of originally four tools for model validation of QSAR models that can be adapted for spectral data resulting in three different tools.

Tool 1 is the permutation test (also referred to as significance test or randomization test). The main idea is to repetitively randomize a certain amount of the *Y* -variables in the training set while the *X*-data stays intact. In each cycle, the full data analysis is carried out on these scrambled data and the *R*^2^ and *Q*^2^ values are recorded. If, in each case, the scrambled data give much lower *R*^2^ and *Q*^2^ values than the original data, it is likely that a real correlation was found.

Tool 2 is CV as explained above. It is a frequently applied and useful approach to model validation. However, CV results may also be misleading. If the validation groups during CV are too small, the model selection is biased. For example, if the number of groups is equal to the sample size, also referred to as leave-one-out, the permutation during the CV is too small and the resulting *Q*^2^ values will approach the *R*^2^ value [114]. In practice, 5–9 subsets are recommended [108]. Additionally, CV might not work for variable selection because only the variables with correlation to the *Y* -data are chosen and this might lead to the selection of *X*-variables with spurious correlations to *Y* [115].

Tool 3 is related to appropriate sample selection and in particular the external validation set. Ideally, an external validation data set should span across the complete design space in an evenly distributed manner. The validation set can also include samples outside the calibrated range for the *Y* -values to improve the confidence in the built model.

We recommend the use all of these tools for model validation to avoid spurious correlations, especially tool 1. When looking at the data published by Walch et al. [18], tools 2 and 3 have been applied, but not tool 1. A permutation test and inclusion of the mAb concentration as *X*-variable could reveal in this example if the concentrations of DNA, HCP, and HMWS were predicted from the mAb concentration. For increasing mAb concentrations, decreasing impurity levels were calculated and vice versa. This may have little to do with actual concentration measurements of these components because the amount of impurities per mAb concentration is not constant for every sample and batch. Especially when a large number of *X*-variables from different sensors are available, extensive variable selection can lead to spurious correlations [79].

### Data fusion

**Fig. 3:**
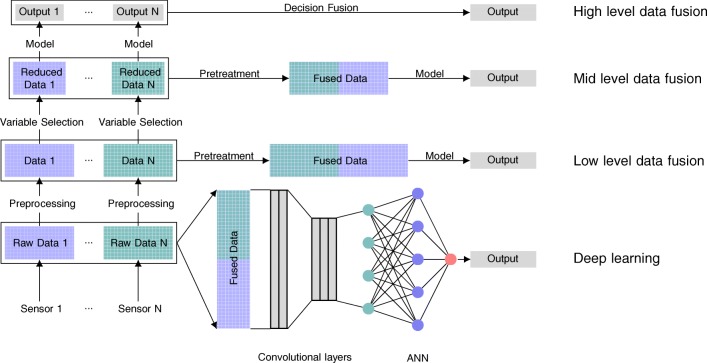
Methodology for model building in low-level, mid-level and high-level data fusion and, additionally, deep learning. Adapted after [24]

When multiple or multimodal sensors are involved in a measurement, different data fusion strategies can be utilized for model building [116]. Data fusion is generally categorized into low-level, mid-level, and high-level data fusion [24, 117, 118]. A general overview is given in Fig. 3. Here, each sensor provides a block of data, which needs to be fused to all the other blocks for analysis. Low-level data fusion concatenates the different raw or preprocessed data blocks and applies an appropriate block-wise pretreatment before model building. This is important because the variables in the blocks typically have different scales. Variables with a higher numeric value would otherwise contribute more to the model. To overcome this problem, unit variance scaling could be performed. Block scaling can be used to multiply the block with an additionally scaling weight to account for the importance of these variables for the prediction of the *Y* -variable [91].

Mid-level data fusion applies variable selection before concatenating the different data blocks to reduce the influence of a large amount of unimportant variables. This can be done by variable selection for the data blocks or by hierarchical multiblock PLS. Hierarchical multiblock PLS is based on the decomposition of the blocks into scores and latent variables. The obtained block scores are subsequently used for PLS model building on the upper level [119]. This increases the interpretability of the model because the relations between the blocks are emphasized due to the upper data level from which the model is built. An additional benefit of hierarchical multi-block PLS is the improved prediction of the block models as they are less sensitive to mild scaling inaccuracies [119].

High-level data fusion is a fusion of the outcome of a model. Therefore, it may rather be termed decision fusion than data fusion [20]. This means that block-scaling is unnecessary and the models can be separately optimized. Methods for decision fusion include different techniques like weighted decision methods, Bayesian inference, Dempster–Shafer inference or fuzzy logic theory [120]. Additionally, if a time dependency is available, state estimation methods like Kalman filters can be used.

Recently, convolutional neural networks (CNNs) have gained momentum in spectral analysis [121–123]. Originally, CNNs were designed to cope with shift and distortion variances for image recognition [124] or speech recognition [125], which is desirable for spectral analysis as well. CNNs are a variant of feed-forward ANNs with additionally convolutional layers to filter the data by weighting the summation of the inputs in windows [126]. The kernels in the convolutional layers are sparsely connected and share weights. CNNs focus rather on local features, which makes them easier to train and interpret, and less prone to overfitting [122]. In higher structural data, pooling layers are used to pool similar features and bring the data in 1D form. For spectral data (already in 1D form), pooling layers are not always used [122].

CNNs are the oldest form of deep learning architectures [127] with multiple levels of nonlinear functions due to many hidden layers. This architecture of CNNs results in a filter ability. Therefore, CNNs can handle raw data, which can make human interference for preprocessing the data unnecessary [124]. However, it has been shown that CNNs work better on preprocessed data similar to how PLS models behave [122]. CNNs are highly flexible and can fit highly nonlinear correlations. Nevertheless, for linear problems, usually linear methods perform better [128].

## Perspectives for the biopharmaceutical downstream process

This final section of the review is intended to give a more abstract view of the present and future of PAT in downstream processing of biopharmaceutical proteins. A special focus is set on different product- and process-related impurities and on how the current approaches could be further integrated towards holistic process monitoring.

**Fig. 4:**
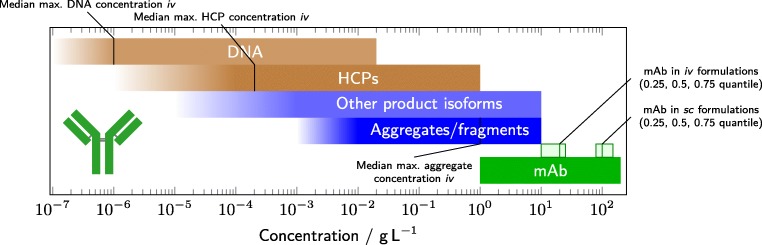
In biopharmaceutical processes, different species need to be monitored in a concentration range spanning many orders of magnitudes. This is illustrated here by the example of mAb processes. Each *horizontal bar* denotes concentration ranges for the major species covered in biopharmaceutical processes. In *green*, the mAb concentration is shown. The *boxes in light green* correspond to the monoclonal antibody concentrations of the marketed mAbs in US for *intravenous (iv)* and *subcutaneous (sc)* administration. Product- and process-related impurities are shown in blue and brown, respectively. Impurity concentration limits as accepted by the regulatory agencies are marked by *black lines* in the corresponding concentration bars

In biopharmaceutical processes, relevant impurities and the product need to be monitored and controlled in a broad concentration range. Figure 4 illustrates this with the typical concentrations occurring during manufacturing of a mAb. Figure 4 also includes the typically maximum allowed impurity concentrations in the drug product. Information on the involved data analysis is provided in the Supplementary Data. Considering the lowest and highest relevant concentrations for both contaminants and mAb, downstream processing is spanning more than seven orders of magnitude of concentration values. Furthermore, each species is a diverse group of substances. For example, the term HCP refers to any protein produced by the host cells in addition to the target product. Thus, HCPs are a very diverse group of proteins which additionally complicates detection or concentration measurements of these contaminants [129, 130]. While the diversity for other species in biopharmaceutical production may not be as extreme as for HCPs, similar arguments hold for DNA, aggregates, fragments, or other product isoforms. The broad concentration ranges in combination with the diversity of the relevant species in downstream processing pose a major challenge for PAT.

In recent publications, implemented in-line soft sensors (spectroscopic methods in combination with chemometrics) achieved limits of detection for aggregate and fragment levels below the concentration limits set by the regulatory agencies for drug products [15, 45, 74]. On a lab scale, the feasibility for measuring these important contaminants with the necessary accuracy was thus demonstrated. Future projects may work towards a closed-loop control of the process steps of interest. Product-related isoforms occur at similar concentrations as aggregates and fragments. Spectroscopic PAT methods are likely to achieve similar limits of detection as long as there is a measurable change in the spectroscopic properties of the isoforms. It seems likely that some processes may also use spectroscopic soft sensors for controlling isoform profiles in the future. However, there also remains a large fraction of isoforms that cannot be distinguished from the product by optical spectroscopy. In such cases, other sensors or control strategies should be evaluated.

For the process-related impurities HCPs and DNA, in-line monitoring may be achievable for early steps in downstream processing, such as capture steps, where the process-related impurity concentrations are still high. During further polishing steps, process-related impurity concentrations are typically by a factor of 10^5^ to 10^10^ lower than the product concentration. To further complicate detection, HCPs are polypeptides and therefore chemically highly similar to the product. DNA is more distinct from the product, but typically also occurs at the lower end of the concentration scale. Based on regulatory guidelines, DNA must be depleted to concentrations approximately 10^7^ times lower than the product concentration. The quantification of HCPs and DNA by optical spectroscopic PAT methods towards the end of the downstream process seems very challenging and probably not achievable in the near future. Furthermore, at the current state of research, a purely measurement-driven approach does not seem practical for monitoring and controlling all critical quality attributes (CQA) in downstream processing in real-time.

Fortunately, there are alternative approaches to monitoring and controlling production processes. For example, model-based predictions of CQAs from observed process parameters have reached an impressive accuracy in a number of studies [131–133]. These studies showed that statistical models can capture a significant amount of the hidden process dynamics and the effects on the CQA of the product while neglecting the actual time evolution of the system. In a next step, it would be interesting to also obtain time-dependent predictions of the process trajectory. Here, mechanistic, hybrid, or empirical models could be applied to predict the underlying system dynamics. As soon as a fast dynamic process model for different CQAs is available, the model could also be leveraged for process control.

While different approaches to process control exist, model predictive control (MPC) is regarded as one of the most important tools in advanced process control [134, 135]. MPC is well established in various industries including refining, petro-chemical, and food applications [136]. MPC is founded on a mathematical model of the process dynamics, i.e., a model which describes the time evolution of the investigated system. To control the process, the model is leveraged by taking current and future process dynamics into account. Based on the model and an objective function, MPC aims to optimize the process performance over a given time frame into the future (the so-called receding horizon) by calculating a number of control actions. At each time step, an optimization is performed to find the optimal control actions. Then, the first calculated control action is applied to the system and the optimization is repeated with the receding horizon reaching one time step further into the future. This approach allows to neglect the future of the process beyond the receding horizon, thus simplifying the control problem. Among the benefits of the MPC framework is also its high flexibility. MPC provides means for accepting input variables, maintains an estimate of the current system state, and predicts the current and future plant outputs. Due to the model-based foundation of MPC, it is particularly well aligned with the motive of quality by design (QbD) of building the quality into the product through product and process understanding (see [137] for an extended discussion).

MPC was already investigated for a number of applications in biopharmaceutical manufacturing. For upstream processing, a number of different MPC schemes have been applied and reviewed [137, 138]. For downstream processing, research focused on the control of continuous chromatography. MPC for multi-column solvent-gradient purification (MCSGP) was developed and advanced in a variety of publications [139–141]. The application of MPC allowed for improved process performance and robust control of the purification processes as demonstrated by in silico studies. The need for reliable PAT was pointed out multiple times to provide feedback to the model. Initial research also exists towards coupling upstream and downstream unit operations in silico for an overall advanced process control [142].

Regarding process- and product-related impurities, MPC and its underlying model could build the basis for controlling CQAs based on inferred sensing of different species. In such a scenario, inferred state variables may track CQAs (e.g., HCP and DNA concentration) within the process which are not directly available from measurements [17, 135]. Based on an in-depth understanding ingrained into a model, MPC provides the ability to control impurities throughout the process, building a so-called Digital Twin of the production. An additional key advantage of MPC is its capability to respect constraints. Thus, the objective function can be adjusted to fulfill the predefined quality metrics. Based on such an approach, manufacturing can be tailored towards real-time release (RTR) [57].

### Electronic supplementary material

(PDF 224 KB)

## Appendix: Calculations of molecular cross sections and absorption coefficients

Equation 2 from Singh et al. [147] was used to convert molar absorption coefficients *𝜖*_*m**o**l**a**r*_ in l mol^− 1^ cm^− 1^ to molecular cross sections *σ* in cm^− 2^ Molecule^− 1^.

4 $$ \frac{\sigma}{\text{cm}^{2}}=3823 \cdot 10^{-24} \frac{\epsilon_{molar}}{\text{l mol}^{-1} \text{cm}^{-1}} $$

The molar absorption coefficient *𝜖*_*m**o**l**a**r*_ was calculated from the absorption coefficient *𝜖* in l g^− 1^ cm^− 1^ and the molar mass *M* in g mol^− 1^ according to Eq. 5.

5 $$ \epsilon_{molar}= \frac{\epsilon}{M} $$

### A.1 Fluorescence

Tryptophan is the most dominant aromatic amino acid in the UV spectrum regarding the absorption coefficient. Its quantum yield is 0.13 [68]. This information was used to convert the absorption coefficient at 280 nm to an emission coefficient.

### A.2 MIR

Typically, mAbs consist mainly of *β*-sheet secondary structure elements [148]. The extinction coefficient of C=O stretch in the amid I band at 1619 cm^− 1^ for *β*-sheet structures is 980 l mol^− 1^ cm^− 1^ [149, 150]. For the calculations, it was assumed that mAbs have roughly 1500 peptide bonds.

### A.3 NIR

NIR band intensities are much weaker than their corresponding MIR fundamentals by a factor of 10 to 100 depending on the order of the overtone [52].

### A.4 Raman

The Raman scatter cross section was calculated from recorded data through comparison of the amid I band with the scattering area of water. The Raman scatter cross section of water 5 × 10^− 30^ cm^− 1^ and a molar concentration of water of 55.5 mol l^− 1^ were used for the calculation [151].

### A.5 Rayleigh scatter

11 nm was used as hydrodynamic diameter of a standard antibody [152, 153]. The Rayleigh scatter cross section was calculated after Cox et al. [154].
