# Supplementary material for: A critical review of recent trends, and a future perspective of optical spectroscopy as PAT in biopharmaceutical downstream processing
Source: Anal Bioanal Chem. 2020 Mar 7;412(9):2047–64. doi: 10.1007/s00216-020-02407-z (PMC7072065; doi:10.1007/s00216-020-02407-z)
Supplement: Supplementary file 1 — (PDF 224 KB) [file 216_2020_2407_MOESM1_ESM.pdf]

Analytical and Bioanalytical Chemistry

Electronic Supplementary Material

A critical review of recent trends, and a future perspective of optical spectroscopy as PAT in biopharmaceutical downstream processing

Laura Rolinger, Matthias Rüdt, Jürgen Hubbuch

A Calculations of Molecular Cross-sections and Absorption Coefficients

Equation 5 from Singh et al. [147] was used to convert molar absorption coefficients ϵ_{molar} in $\mathrm{L\,mol^{-1}\,cm^{-1}}$ to molecular cross- sections σ in $\mathrm{cm^2\,Molecule^{-1}}$.

$$\frac{\sigma}{\mathrm{cm}^2} = 3823 \cdot 10^{-24} \frac{\epsilon_{molar}}{\mathrm{Lmol^{-1}cm^{-1}}} \tag{7}$$

The molar absorption coefficient ϵ_{molar} was calculated from the absorption coefficient ϵ in $\mathrm{L\,g^{-1}\,cm^{-1}}$ and the molar mass M in $\mathrm{g\,mol^{-1}}$ according to Equation 8.

$$\epsilon_{molar} = \frac{\epsilon}{M} \tag{8}$$

A.1 Fluorescence

Trypthophan is the most dominant aromatic amino acid in the UV spectrum regarding the absorption coefficient. It's quantum yield is 0.13 [68]. This information was used to convert the absorption coefficient at 280 nm to an emission coefficient.

A.2 MIR

Typically, mAbs consist mainly of β-sheet secondary structure elements [148]. The extinction coefficient of C=O stretch in the amid I band at $1619\,\mathrm{cm^{-1}}$ for β-sheet structures is $980\,\mathrm{L\,mol^{-1}\,cm^{-1}}$ [149], [150]. For the calculations, it was assumed, that mAbs have roughly 1500 peptide bonds.

A.3 NIR

NIR band intensities are much weaker than their corresponding MIR fundamentals by a factor of 10 to 100 depending on the order of the overtone [52].

A.4 Raman

The Raman scatter cross-section was calculated from recorded data through comparision of the amid I band with the scattering area of water. The Raman scatter cross-section of water $5 \times 10^{-30}\,\mathrm{cm^{-1}}$ and a molar concentration of water of $55.5\,\mathrm{mol\,L^{-1}}$ were used for the calculation [151].

A.5 Rayleigh scatter

11 nm was used as hydrodynamic diameter of a standard antibody [152], [153]. The Rayleigh scatter cross-section was calculated after Cox et al. [154].

B Analysis of mAb and Impurity Concentrations

This document gives an overview of how literature data was compiled to yield all the necessary information for the concentration bar plot of mAbs and impurities.

B.1 Main product mAb

- Lower limit 1 g/L [1].
- Upper limit 200 g/L [2].
- The 25%, 50%, and 75% quantile of the mAb concentration in subcutaneous (sc) and intravenous (iv) formulations was calculated from the CDER Billable Biologic Product List [3].

[1]:
```
import numpy as np

# mab concentrations iv
quantiles_conc_mab_formulation_iv = {0.25: 10,
                                     0.5: 20,
                                     0.75: 25} # g/L
median_conc_mab_formulation_iv = quantiles_conc_mab_formulation_iv[0.5]

# mab concentrations sc
quantiles_conc_mab_formulation_sc = {0.25: 78.75,
                                     0.5: 100,
                                     0.75: 150} # g/L
median_conc_mab_formulation_sc = quantiles_conc_mab_formulation_sc[0.5]

# mab concentration range
conc_dict = {'mab': [1,200]}
```

B.2 Product-related impurities

According to Chon et al., the maximal concentrations of aggregates at the end of production should typically be below 5% of the mAb concentration, given the median mAb concentration of 20 g/L iv, this corresponds to 1 g/L [4]. Typical minimal concentrations are, however, much lower. Aggregates are often quantified by SEC down to 1% of mAb concentration. 1% of minimal mAb concentration corresponds to 0.01 g/L. In the case of a highly clipped/aggregating mAb, concentrations of 10 g/L aggregate/fragments may be reached in an intermediate step.

Other product-related impurities, such as charge isoforms, misfolds, etc. may be interesting to even lower concentrations. For example, Wang et al. reported detection levels of product variants in the hinge region in the range of 0.3% to 6.2% [5]. The upper limit was chosen in accordance to the reasoning for aggregates/fragments as 10 g/L.

[2]:
```
conc_dict['product isoform'] = [0.003*conc_dict['mab'][0], 0.
 ↪064*median_conc_mab_formulation_iv]
conc_dict['aggregates'] = [0.001,10]
```

B.3 Process-related impurities

B.3.1 HCPs

According to Chon et al., the common industry specification is that HCP levels need to be reduced to below 100 ppm at the final stage of the process [4]. Based on the iv mAb concentration,

a median maximal HCP concentration can be calculated.

For typical maximal and minimal HCP concentrations in mAb DSP, literature data was used [6], [7]. Of the used HCP concentrations, the median was calculated to get typical maximum and minimum boundaries.

[3]:
```
# Calculation of maximal occuring HCP concentrations
hcp_levels_high = np.array([864616, 151125, 103304, 153500,488854]) # as␣
↪listed in references
median_hcp_high = np.median(hcp_levels_high)
conc_hcp_levels_high = median_hcp_high * conc_dict['mab'][0]*1e-6
print('Median reported HCP levels at early process stage: \n{} g/L'.
↪format(conc_hcp_levels_high))

# Calculation of minimal occuring HCP concentrations
hcp_levels_low = np.array([4.7,3.2,8.5,18.1,5,12]) # as listed in references
median_hcp_low = np.median(hcp_levels_low)
conc_hcp_levels_low = median_hcp_low * median_conc_mab_formulation_iv * 1e-6

print('Median reported HCP levels at late process stage: \n{} g/L'.
↪format(conc_hcp_levels_low))
conc_dict['hcp'] = [conc_hcp_levels_low, conc_hcp_levels_high]
```

```
Median reported HCP levels at early process stage:
0.1535 g/L
Median reported HCP levels at late process stage:
0.000135 g/L
```

B.3.2 DNA

For the DNA concentration, reference data was obtained for the allowed level per dose. To convert the values to concentrations, the median iv mAb concentration as well as the median dose (both optained from CDER report) were used. The World Health Organization (WHO) has recommended that DNA levels in biological products produced by genetically modified organisms in immortal cell lines be consistently reduced to levels below 10 ng/dose (while still taking into account other factors) [8].

Estimated typical DNA content in HCCF range from 7900 ng/mg to 15000 ng/mg according to Butler et al. [9].

[4]:
```
mab_dose = 0.2
volume_dose = mab_dose / median_conc_mab_formulation_iv
max_allowed_dna = 10/(volume_dose)*10**-9

max_conc_dna = 15000*1e-6*conc_dict['mab'][0]

conc_dict['dna'] = [max_allowed_dna, max_conc_dna]

print('Max allowed DNA as concentration in formulated drug: {} ng/L'.
↪format(round(max_allowed_dna*10**9)))
print('Typical DNA concentration in HCCF: {} g/L'.format(max_conc_dna))
```

```
Max allowed DNA as concentration in formulated drug: 1000 ng/L
Typical DNA concentration in HCCF: 0.015 g/L
```

References

[1] E. Pacis, N. Vijayasankaran, J. Li, M. Gawlitzek, A. Amanullah, and F. Li, “Systematic approaches to develop chemically defined cell culture feed media,” BioPharm International, vol. 23, no. 11, 2010.

[2] J. Rucker-Pezzini, L. Arnold, K. Hill-Byrne, T. Sharp, M. Avazhanskiy, and C. Forespring, “Single pass diafiltration integrated into a fully continuous mab purification process,” Biotechnology and bioengineering, vol. 115, no. 8, pp. 1949–1957, 2018.

[3] Center for Drug Evaluation and Research (CDER). (Jun. 2019). CDER therapeutic biologic products, [Online]. Available: https://www.fda.gov/media/76650/download (visited on 01/08/2019).

[4] J. H. Chon and G. Zarbis-Papastoitsis, “Advances in the production and downstream processing of antibodies,” New biotechnology, vol. 28, no. 5, pp. 458–463, 2011.

[5] D. Wang, C. Wynne, F. Gu, C. Becker, J. Zhao, H.-M. Mueller, H. Li, M. Shameem, and Y.-H. Liu, “Characterization of drug-product-related impurities and variants of a therapeutic monoclonal antibody by higher energy c-trap dissociation mass spectrometry,” Analytical chemistry, vol. 87, no. 2, pp. 914–921, 2015.

[6] M. Vanderlaan, W. Sandoval, P. Liu, J. Nishihara, G. Tsui, M. Lin, F. Gunawan, S. Parker, R. M. Wong, J. Low, et al., “Hamster phospholipase b-like 2 (plbl2): A host-cell protein impurity in therapeutic monoclonal antibodies derived from chinese hamster ovary cells,” BioProcess International, vol. 13, no. 4, pp. 18–29, 2015.

[7] H. Liu, W. Riches, M. Hylands, S. Zhang, and M. Byrne, “Hcp antigens and antibodies from different cho cell lines,” BioProcess International, 2017.

[8] World Health Organization (WHO), “Annex 3: Recommendations for the evaluation of animal cell cultures as substrates for the manufacture of biological medicinal products and for the characterization of cell banks, replacement of annex 1 of who technical report series, no. 878,” 978, 2013.

[9] M. D. Butler, B. Kluck, and T. Bentley, “Dna spike studies for demonstrating improved clearance on chromatographic media,” Journal of Chromatography A, vol. 1216, no. 41, pp. 6938–6945, 2009.
